# Society for Cardiovascular Magnetic Resonance incoming president’s page : New frontiers in advancing cardiovascular magnetic resonance to improve cardiovascular health

**DOI:** 10.1016/j.jocmr.2026.102696

**Published:** 2026-02-09

**Authors:** Vanessa M. Ferreira

**Affiliations:** University of Oxford Centre for Clinical Magnetic Resonance Research (OCMR), Division of Cardiovascular Medicine, Radcliffe Department of Medicine, University of Oxford, The John Radcliffe Hospital, Oxford, Oxfordshire, United Kingdom

As the incoming President of the Society for Cardiovascular Magnetic Resonance (SCMR), it is with great excitement and optimism that I take on the responsibility in this important leadership role. I would like to express my gratitude to the SCMR membership for their support and trust in me to continue the great legacy that we have achieved together, and to bring the Society to new heights. My vision for the year ahead, and beyond, is to explore new frontiers in advancing CMR to improve cardiovascular health on multiple levels—whether geographically, scientifically, clinically, or in the way we work together as a community. We are in a unique position, with knowledge and expertise in CMR as a powerful advanced multiparametric imaging modality, to address important clinical questions and to pursue scientific discoveries for the betterment of cardiovascular health globally.

CMR is an imaging gold standard for assessing cardiac structure, function and tissue characterization, with excellent multiparametric capabilities, including assessment of myocardial perfusion and blood flow. However, the access and clinical adoption of CMR remain challenges, with significant variations globally. The reasons are multifactorial, including relative expense in commissioning and maintaining a CMR service, the need for specialized personnel, challenges in accessing CMR training in some regions, relatively long scan times and waiting times, financial issues surrounding billing and reimbursement, and at times, local challenges in who should perform and interpret CMR. Although CMR is driven by technical innovation, translation of novel and clinically validated methods often take years and even decades to become a clinical product to benefit patients; collaboration with industry partners is essential. For CMR methods to become recognized in international clinical guidelines, robust evidence demonstrating their clinical value is needed, ideally in the form of Level A evidence (data derived from multiple, high-quality randomized controlled trials or systematic reviews/meta-analyses of such trials), which are currently sparse for CMR. Clearly, we, as a community, need to work together in new ways to address these issues, for CMR to flourish as a mainstream imaging modality in routine clinical practice worldwide.

In Iine with SCMR’s Strategic Plan and Global Expansion Program, and further to SCMR’s recent activities in Latin America, one of my goals is to increase SCMR’s collaboration and education offerings in Asia, Eastern Europe and beyond, in collaboration with colleagues and sister societies in these regions. In the recent years, SCMR has delivered a number of successful Level I and Advanced Courses in these regions, such as India, Hong Kong and Lithuania. These activities have been met with great enthusiasm locally and attracted attendees from further afield. It is anticipated that SCMR will continue to expand its presence globally to deliver on our mission to provide CMR education and engagement worldwide.

Ready access to a local clinical CMR service remains a global challenge, and this must be addressed for the routine clinical adoption of CMR and its sustained growth. High upfront and maintenance costs impose a high entry threshold, especially for areas with less developed healthcare infrastructures. Often, it is insufficient for novice CMR professionals to only attend courses or short-term fellowships if aiming to set up a clinical CMR service at their home institution. While training of CMR-reporting physicians and technologists is essential, practical, on-the-ground support to start a CMR service can be invaluable. This may include how to build a business case to commission a clinical CMR service, on-site proctoring, practical small group hands-on scanning tutorials led by experienced CMR practitioners undertaken at the home MRI unit, sharing of “tips and tricks,” standard operating procedures and expertise on how to manage day-to-day workflow. A support network for exchanging experiences and practical solutions to daily issues can foster a constructive environment that ultimately improves the quality of a CMR service. There are multiple areas for further development, including more affordable hardware (e.g., low-field CMR), AI-driven solutions (semi-/automated and fast scanning protocols and reporting tools), and widening access to CMR training. These are within the remit of SCMR’s missions, and there are both existing opportunities as well as room for further work.

The field of CMR is in need of more clinical trials and Level A evidence (randomized controlled trials or systematic reviews/meta-analyses of such trials), to support its inclusion in international guidelines and its use in routine clinical practice. To this end, new initiatives and investments will be explored, aiming to facilitate and support multicenter, multivendor clinical trials that generate robust evidence to definitively advance the position of CMR in the management of cardiovascular diseases. This will be undertaken together with the SCMR Board, Executive Committee, the Clinical Trials Committee, and our industry partners, including via the Corporate Advisory Board (CAB). New schemes and calls for proposals from experienced CMR trialists to address this need are anticipated, to further assess how SCMR may facilitate and support external applications for funding.

For CMR to thrive, it is imperative that we work together as a community, including radiologists, cardiologists, congenital/pediatric CMR imagers, MR scientists and technologists. In particular, SCMR advocates for a competency-based curriculum for CMR, which outlines the key competencies required for Level II and III CMR practitioners, whether trainees come from a radiology or cardiology background. The SCMR periodically highlights successful examples of clinical CMR services in which radiologists and cardiologists work constructively and synergistically. One such recent exemplary achievement is a sustained effort by Dr Tomas Lapinskas—a cardiologist and President of the Lithuanian Society of Cardiology. Dr Lapinskas not only proactively sought training in CMR when there was no CMR provision in Lithuania, but also brought CMR back to his country, and worked together with his radiology colleagues to appeal to the government to commission CMR as a clinical service in which both radiologists and cardiologists can report CMR independently. SCMR strongly supports such collaborative approaches, and had, with Dr Lapinskas, successfully launched an SCMR Level I course in Lithuania in 2025 with overwhelming reception, to be followed by an SCMR Stress Perfusion Course in 2026. There have been other similarly successful examples of cardiologists and radiologists working effectively together to promote accessibility to CMR. It is expected that senior leaders in SCMR will demonstrate evidence of collaborative interdisciplinary working practices, including when running for SCMR elections and applying for SCMR awards, where applicable. Interdisciplinary and collaborative working models ultimately promote higher quality and improved access to CMR for better patient care.

The CMR community constantly strives to innovate new ways to image the cardiovascular system for clinical use. However, bringing new MRI methods from bench to bedside currently is mired by an unsatisfactorily slow process, sometimes taking decades, before methods that are clinically well-validated become a clinical product. To this end, the SCMR Efficiency Task Force (led by past-president Michael Markl) had outlined five focus areas, of which one was “*Future Directions, Imaging Technology Innovations*”. As its lead, I, together with other task force members, have put forth recommendations as follows: (1) to identify main CMR challenges that could be addressed and/or be solved by new imaging techniques; (2) to develop SCMR-endorsed documents for research-to-clinical translation of imaging techniques (including minimal requirements for performance, quality, level of evidence, metrics of success, etc.); (3) that SCMR could promote new CMR techniques that have reached a certain level of evidence; (4) to develop strategy for collaboration with MRI systems vendors to integrate new imaging techniques onto MRI scanners, and; (5) to develop guidance for the use of research sequences in the clinical setting.

To that end, a pre-conference workshop, “*Turning Innovations into Action*,” will take place prior to the Opening Plenary of the 2026 SCMR Annual Scientific Sessions, aiming to examine how research innovations can be efficiently translated into clinical practice. A newly launched SCMR Innovations Task Force will also aim to bridge the gaps between scientific discovery and patient care, and to shorten the innovation-to-commercialization timeline. A closer collaboration with our industry partners could help identify mutually beneficial and synergistic areas of work, to fast-track CMR and harness its capabilities as a powerful clinical tool that should be widely available in mainstream clinical practice worldwide.

The growth of SCMR continues to soar, whether in membership numbers, its financial health or global reach. As of January 2026, SCMR has nearly 3600 active members, and over 21,600 members within the SCMR community. As announced in 2025, the SCMR Board had taken the historical and strategic decision to transition into a fully self-managed organization from October 1, 2025. Operating as a standalone association will enable us to be more responsive to the evolving needs of our community, to direct resources more strategically toward our priorities, and to reinforce the unique identity and culture of SCMR. We now have an excellent SCMR Staff Team, and we have exciting plans to continue to develop SCMR as the largest leading international professional society dedicated to CMR. It is anticipated that we will expand this team accordingly to serve our membership.

Together, as a multidisciplinary CMR community, we will continue to deliver on SCMR’s mission to improve global cardiovascular health by leveraging the advantages of CMR. We will accomplish our mission through innovation, education, advocacy, networking, research, and clinical excellence. Collectively, we have a bright future where CMR, a sophisticated imaging solution with multiparametric capabilities for assessing a broad range of cardiovascular conditions, will become widely accessible, to benefit patients and their care, ultimately bettering cardiovascular health for all.

## Declaration of competing interests

Vanessa Ferreira reports a relationship with GE Healthcare that includes speaking and lecture fees, investigator-sponsored research and travel reimbursement. Vanessa Ferreira reports a relationship with Circle Cardiovascular Imaging, Canon Medical Systems and Edicate by Medica that includes speaking and lecture fees.

